# Social connection and depression: an umbrella review of meta-analyses assessing the magnitude of risk and protection

**DOI:** 10.1192/j.eurpsy.2023.896

**Published:** 2023-07-19

**Authors:** M. Pettorruso, R. Collevecchio, F. Zoratto, B. Collacchi, M. Boffa, M. Santorelli, M. Clerici, G. Martinotti, M. Borgi

**Affiliations:** 1 Department of Neuroscience, Imaging and Clinical Sciences, “G. d’Annunzio” University of Chieti-Pescara, Chieti; 2 Center for Behavioural Science and Mental Health, Istituto Superiore di Sanità, Rome; 3 School of Medicine and Surgery, University of Milano-Bicocca, Milan, Italy

## Abstract

**Introduction:**

Depression is a severe mental disorder with an estimated 3.8% of the population affected, representing a leading cause of disability worldwide. Being linked to reduced quality of life and individual functioning, medical morbidity and mortality, depression has a huge social and economic impact. A wide range of potentially modifiable factors for depression has been identified. Among these, social factors (e.g. support/engagement) appear to play a major role in the emergence and severity of depression.

**Objectives:**

We aimed at providing a quantitative synthesis of the consistency and magnitude of the association between measures of social connection and depression. Social connection included both quantitative (i.e. existence/absence of social relationships) and functional (i.e. support provided) measures of social relationships, as well as measures focusing on social inequalities related to participation in community spaces/activities (i.e. social discrimination).

**Methods:**

We searched PubMed, PsycINFO, Cochrane Library and EMBASE. The strength of the association between exposure factors (social measures) and depression was extracted and equivalent odds ratios were computed to compare the strength of the effect sizes among meta-analyses. The quality of each review was assessed using AMSTAR-2.

**Results:**

As a result of the selection process 47 studies were included in the umbrella review. Social support was found to have a protective role on depression, with an observed moderate/strong effect in peripartum population and a weaker effect in clinical populations (e.g. AIDS/HIV patients). A moderate association between stigma/discrimination and depression emerged in clinical populations (e.g. epilepsy, mental illness, post-stroke), while a weaker effect was found in (ethnic) minorities. There are still few studies quantitatively investigating the link between depression and other social measures (e.g. community connectedness).

**Conclusions:**

Our findings align with the literature on social connection and mental health, confirming the role of social determinants in the emergence and severity of depression, particularly in the case of vulnerable populations. Social factors emerge as important modifiable targets in the context of depression prevention. Efforts to counteract disconnection at the societal and individual levels and to reduce stigma should be central to an effective depression prevention agenda.

**Disclosure of Interest:**

None Declared

